# Effects of stapes surgery prosthesis type on hearing outcome, post-operative dizziness and benzodiazepine use

**DOI:** 10.1017/S0022215125103575

**Published:** 2025-12

**Authors:** Sarah G. Wilkins, Rema Shah, Caroline Valdez, Devesh Malik, Sidharth Tyagi, Douglas Hildrew, Nofrat Schwartz

**Affiliations:** 1 Yale University School of Medicine, New Haven, CT, USA; 2 Division of Otolaryngology-Head and Neck Surgery, Department of Surgery, Yale University School of Medicine, New Haven, CT, USA

**Keywords:** conductive hearing loss, dizziness, hearing outcomes, otosclerosis, stapedectomy, stapedotomy

## Abstract

**Objective:**

Investigate the impact of surgical method on hearing outcomes and complication rates after otosclerosis surgery.

**Methods:**

Records of patients more than 18 years old who underwent otosclerosis surgery were reviewed to identify prosthesis type, surgical approach, post-operative dizziness, overnight admissions and hearing outcomes.

**Results:**

A total of 132 stapedotomies were performed with McGee pistons and 144 stapedectomies were performed using ribbon loops. No sensorineural hearing loss was noted with both techniques. Stapedotomy patients had a statistically larger improvement in speech reception thresholds, but there was no significant difference in air–bone gap closure between the two methods. 3.7 per cent of stapedotomy patients experienced post-operative dizziness, which was not significantly different the 7.6 per cent dizzy after stapedectomy (*p* = 0.2037). Diazepam was prescribed for dizziness in 90.9 per cent (10/11) of dizzy patients with ribbon loops and 0 per cent of those (0/5) with McGee pistons (*p* = 0.0018).

**Conclusion:**

Both approaches yielded similarly good air–bone gap closure and were found to be safe and effective with low post-operative dizziness.

## Introduction

Otosclerosis is a common aetiology for hearing loss in adults, causing more than 20 per cent of the cases of conductive hearing loss.^1^ Treatment for this disease has evolved over the years to include two main surgical techniques: stapedotomy and stapedectomy.^2^ The prosthesis used as part of the surgery has not only evolved but has also diversified to include a variety of types and materials, such as Bucket Handles, pistons and wire loops.^3^ Choice of prosthesis is highly dependent on surgical experience and technical approach,^4^ and surgeons are very unlikely to change their prosthesis and technique of choice, making it difficult for authors to compare outcomes between various prosthesis types and surgical approaches. As a result, few studies exist thus far comparing prostheses in a head-to-head manner. Nevertheless, it is important to attempt to characterize the advantages and risks of different surgical approaches and prosthesis types. While previous studies show that 94 per cent of patients obtain good hearing outcomes with an improved air–bone gap (ABG) less than 10 dB and with a significantly low risk of deafness (1-2%),^5^^,^^6^ many patients are still at risk for several post-operative complications, such as vertigo, tinnitus, prosthesis displacement and facial nerve injury.^7^ Vertigo, specifically, has been characterized as a particularly common and distressing aspect of stapes surgery, with some studies reporting incidence in upwards of 73 per cent of cases.^8^ Thus, given its widespread nature, assessing whether there are associations with prosthesis type is critical.

To alleviate the discomfort of vertigo, patients are often prescribed benzodiazepines as vestibular suppressants.^9^ Additionally, given their anxiolytic effects, they also have the ability to reduce the anxiety associated with acute vertigo.^10^ However, the severe side effect profile of this drug and its risk for abuse require caution and vigilant monitoring.^11^ Given that benzodiazepine use in the adult population has increased, with misuse accounting for 20 per cent of use overall,^12^ efforts to understand prescribing patterns in post-operative settings in order to reduce and replace its need is necessary for public health and safety.

To our knowledge, this is the first paper that investigates the relationship between stapes prosthesis type, immediate post-operative dizziness and the need for post-operative controlled substances based on prosthesis type and type of surgery. In conjunction, we also assessed the correlation between prosthesis type and hearing outcomes to help direct patient counselling on surgical options for otosclerosis.

## Materials and methods

This study was deemed exempt by the Human Research Protection Program Institutional Review Board at our institution. Retrospective chart review from 2013 to 2022 was performed at our institution. Patients 18 years or older with a diagnosis of otosclerosis who underwent stapedectomy or stapedotomy were eligible for study inclusion. The Joint Data Analytics Team at our institution identified patients using the CPT codes 69660 and 69662. Selected patients were verified manually through electronic medical record (EMR) review. EMR review was also used to identify patient demographic information such as age at admission, sex, race and BMI. Additional variables such as prosthesis type, surgical approach and incidence of inpatient admission were also gathered. Post-operative dizziness was evaluated by identifying patients who required admission in the first post-operative days or that required prescription of either meclizine or diazepam for dizziness. Diazepam prescription indicated more severe dizziness compared to patients who only received meclizine. Hearing outcomes were evaluated from measurements of pre- and post-operative air and bone pure tone thresholds as well as speech reception thresholds (SRT) and word recognition score (WRS).

### Surgical method

The operations were performed by three experienced surgeons. Two performed stapedotomies using the McGee piston (MP) prosthesis and one performed stapedectomies using ribbon loop (RL) prosthesis. One surgeon performed the majority of the stapedotomy cases (∼90%). The stapedotomy approach used a CO2 laser to complete a stapedotomy in a rosette fashion with temporalis fascia used to seal the oval window. The surgeon who performed a minority of stapedotomy cases also used a CO2 laser to complete a stapedotomy in a rosette fashion but used a fat graft from the lobule to seal the oval window. The stapedectomy approach used a straight pick to make a hole in the stapes, an angled hook to remove the entire foot plate in two pieces and a perichondrium graft to seal the oval window. The typical protocol for peri-operative audiograms at our institution is as follows: pre-operative audiogram one to two months before surgery, post-operative audiogram approximately three months after surgery.

### Statistical analysis

Analyses were conducted using SPSS v.28 and GraphPad Prism v 10.3.1. Nominal data were analysed by means of Fisher’s exact test and mean values for audiology and demographic data were compared using unpaired T tests. A value of *p* ≤ 0.05 was considered indicative of statistical significance. Continuous variables are represented with the mean and standard deviation.

## Results and analysis

Our cohort included a total of 276 stapes surgeries for otosclerosis that treated a total of 243 unique patients. A total of 132 surgeries used MP prostheses (used inter-changeably with stapedotomy), and 144 used RL prostheses (used inter-changeably with stapedectomy). Of the cohort, one stapedotomy patient underwent initial, followed by revision surgery, 18 patients had bilateral surgeries and one patient had three separate surgeries. Five other stapedotomy patients had revision surgery only. Three stapedectomy patients underwent initial, followed by revision surgery and 10 patients had bilateral surgeries. Seven other stapedectomy patients had revision surgery only.

Of our 243-patient cohort, 40.3 per cent (n = 98) were male and 77.3 per cent (n = 188) were white. Between the two groups, there was no significant difference in average age or body mass index (BMI). There was a significant difference between the racial makeup of the two patient cohorts, with a higher proportion of white patients who received a stapedectomy. Full demographic details can be found in Table 1.

**Table 1.** Demographic information by prosthesis type

	Prosthetic type		
	Stapedectomy (n = 133 patients)	Stapedotomy (n = 110 patients)	p Value
**BMI**	27.2 ± 5.5	27.5 ± 5.4	0.71
**Age**	51.3 ± 12.3	50.3 ± 12.5	0.53
**Gender**			0.69
Male	52	46	
Female	81	64	
**Race**			**0.0026**
White	114	74	
Black	3	5	
Asian	2	10	
Other/Not Listed/Prefer Not to Say	14	21	

**Figure 1.**
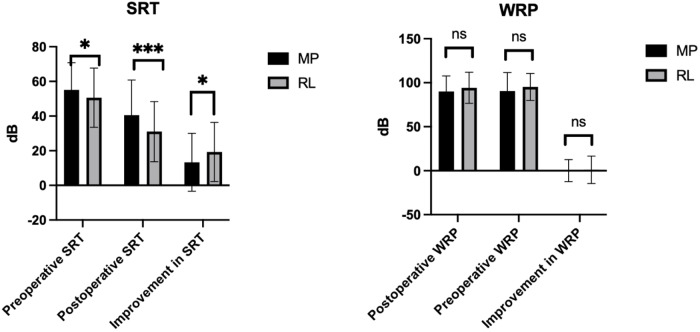
SRT and WRS comparisons for MP and RL patients.

**Figure 2.**
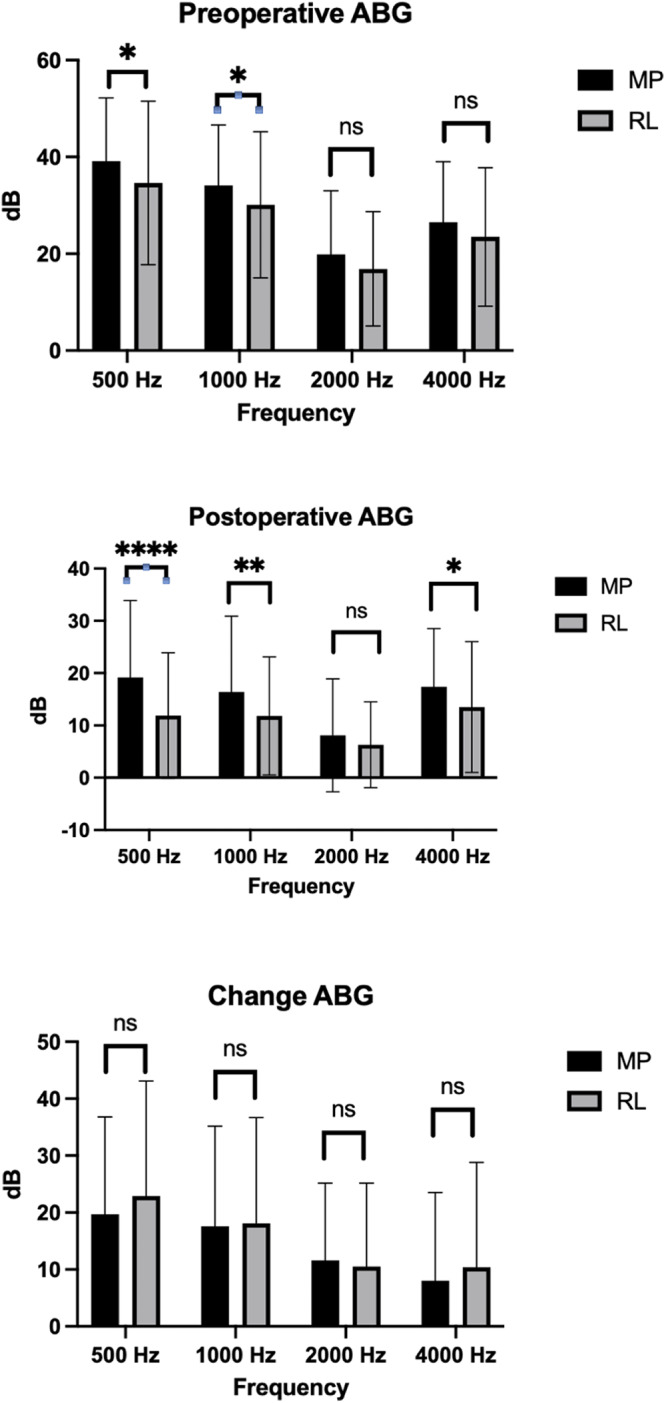
Preoperative ABG, Postoperative ABG, and Improvement in ABG comparisons for MP and RL patients.

Assessment of SRT levels demonstrated that patients with stapedectomy had a significantly better post-operative threshold compared to patients with stapedotomy (31.0 ± 17.4 dB vs. 40.5 ± 20.3 dB, *p* = 0.0003). While the stapedectomy patients also had a significantly better pre-operative threshold (50.6 ± 17.1 vs. 55.1 ± 15.7, *p* = 0.0457), the ∆SRT was still significantly different between the two groups (19.3 ± 17.1 vs. 13.3 ± 16.7, *p* = 0.0164) (Figure 1, Table 2). Regarding the ABG, there were statistically significant differences between the pre-operative ABGs of the stapedotomy and stapedectomy groups at the 500 Hz frequency (MP = 39.1 ± 13.1 vs. RL = 34.6 ± 16.9, *p* = 0.0278) and the 1000 Hz frequency (MP = 34.1 ± 12.5 vs. RL = 30.1 ± 15.1, *p* = 0.0327). Stapedectomy patients had a lower post-operative ABG that was statistically significant at three out of the four frequencies (500 Hz: MP = 19.2 ± 14.7 vs. RL = 11.9 ± 12.0, *p* = 0.001; 1000 Hz: MP = 16.4 ± 14.5 vs. RL = 11.8 ± 11.3, *p*  = 0.0075; 4000 Hz: MP = 17.4 ± 11.1 vs. RL = 13.5 ± 12.5, *p*  = 0.017). However, the ∆ABG was not statistically different between the two groups at any of the four tested frequencies. Full ABG data can be found in Figure 2 and Table 2. With regards to hearing outcomes, the word recognition score (WRS) remained stable across all prosthesis types.

**Table 2.** Audiology variables by prosthesis type

	Prosthetic type		
Audiology variables (Average ± Standard deviation)	McGee piston	Ribbon loop	p Value
Preoperative SRT (dB)	55.1 ± 15.7	50.6 ± 17.1	**0.0457**
Postoperative SRT (dB)	40.5 ± 20.3	31.0 ± 17.4	**0.0003**
Improvement in SRT (dB)	13.3 ± 16.7	19.3 ± 17.1	**0.0164**
Preoperative WRS (%)	90.1 ± 17.7	94.2 ± 17.7	0.0875
Postoperative WRS (%)	90.6 ± 20.9	95.2 ± 15.3	0.0672
Improvement in WRS (%)	0.0 ± 12.5	1.0 ± 15.6	0.6334
Preoperative ABG @ 500 Hz (dB)	39.1 ± 13.1	34.6 ± 16.9	**0.0278**
Preoperative ABG @ 1000 Hz (dB)	34.1 ± 12.5	30.1 ± 15.1	**0.0327**
Preoperative ABG @ 2000 Hz (dB)	19.9 ± 13.1	16.9 ± 11.8	0.0686
Preoperative ABG @ 4000 Hz (dB)	26.5 ± 12.5	23.5 ± 14.3	**0.0958**
Postoperative ABG @ 500 Hz (dB)	19.2 ± 14.7	11.9 ± 12.0	**0.0001**
Postoperative ABG @ 1000 Hz (dB)	16.4 ± 14.5	11.8 ± 11.3	0.0075
Postoperative ABG @ 2000 Hz (dB)	8.1 ± 10.8	6.3 ± 8.2	0.1661
Postoperative ABG @ 4000 Hz (dB)	17.4 ± 11.1	13.5 ± 12.5	**0.017**
∆ABG @ 500 Hz (dB)	19.7 ± 17.1	22.9 ± 20.2	0.2207
∆ABG @ 1000 Hz (dB)	17.6 ± 17.6	18.1 ± 18.6	0.84
∆ABG @ 2000 Hz (dB)	11.6 ± 13.6	10.5 ± 14.7	0.5835
∆ABG @ 4000 Hz (dB)	8.0 ± 15.5	10.4 ± 18.4	0.3216

Focusing on post-operative dizziness, dizziness was noted in 3.8 per cent (n = 5) of the stapedotomy cases with the MP prosthesis, which was not significantly different than the 7.6 per cent (n = 11) of those dizzy after stapedectomy with RL prosthesis (*p* = 0.2037). Overall, low overnight admission for dizziness was noted at 1.4 per cent, which included three patients with RL and one with MP prostheses. When a medication was prescribed to alleviate symptoms of dizziness, diazepam was prescribed in 10 out of 11 cases and meclizine was prescribed once after stapedectomy. All five stapedotomy patients were prescribed meclizine (*p* = 0.0014). None of the patients in our cohort required long-term controlled substances and patients did not receive prescriptions for dizziness concerns beyond one week post-operatively.

## Discussion

Our study evaluates the combination of how different surgical approaches and prosthesis types affect hearing outcomes and post-operative dizziness in a large surgical cohort of patients with otosclerosis. When comparing stapedectomy vs. stapedotomy patients, stapedectomy patients had improved SRTs, though both groups had a similar closure of the ABG after surgery. Stapedectomies (RL) were associated with an increased use of diazepam for post-operative dizziness when compared with stapedotomies (MP).

### Hearing outcomes

Differential outcomes and complication rates between stapedotomies and stapedectomies for otosclerosis have been the subject of much debate in the field. For example, Handke *et al.* demonstrated that audiological outcomes after stapes surgery depend on the prosthesis type and are frequency specific, with crimp prostheses showing significantly better ABG closure at 0.5 kHz.^13^ Moreover, as outlined by the 2018 review by Cheng and colleagues,^14^ an important work by House *et al*. showed that ABG closure was similar between the surgical approaches, other than at high-tone frequencies (4000 Hz). The study by House *et al*. was particularly robust as they included a subset of patients with bilateral pathology who had undergone a stapedotomy on one ear and a stapedectomy on the other.^15^ At least four subsequent works (as reviewed by Cheng and colleagues), additionally found that stapedotomy resulted in better closure of the ABG, particularly at higher frequencies.^14^ However, other data has shown the two outcomes to be similar, such as a recent work by Teixeira-Marques *et al*.^16^ as well as Odat *et al*.^17^

In our cohort, we found no significant difference in the average ABG improvement between the two surgical techniques, suggesting that both stapedotomy and stapedectomy were equally effective in closing the ABG and achieving good hearing outcomes. Furthermore, our data indicates that the stapedectomy approach resulted in better SRT and greater SRT improvement. In contrast to previous studies, our findings do not support the notion that stapedotomy is superior to stapedectomy in terms of hearing outcomes; rather, the two approaches appear at least equivalent.

Interestingly, the stapedectomy ABGs were generally lower both pre- and post-operatively, signalling that these patients had more mild disease at the time of surgery. It is unclear whether milder disease at date of surgery would impact potential post-operative improvement. Regardless, this finding raises an important point: surgeon preference is a significant factor when comparing these two groups; not only do surgeons typically have a methodological preference, but they also have different operative thresholds for hearing loss severity when electing to pursue surgery.

While there were no differences between the stapedotomy and stapedectomy groups in terms of age, BMI and gender, significant differences existed in terms of the racial makeup of each cohort. It is known that otosclerosis is more prevalent in white individuals,^18^ and there is not a large body of data to our knowledge exploring the impact of race on hearing outcomes in regards to otosclerosis patients. Thus, the potential impact of the differences in racial makeup of the respective cohorts remains unknown. Our data support the conclusion that both stapedotomy and stapedectomy lead to significant improvements in hearing outcomes for patients with otosclerosis. Both procedures are viable surgical options, with the choice often depending on the surgeon’s expertise and institutional preference. We affirm that both stapedotomy and stapedectomy are safe, effective and reliable methods for improving hearing in otosclerosis patients.

### Post-operative dizziness and medication regimens

When investigating the differences in immediate post-operative dizziness, previous authors have advocated for the use of stapedotomies over stapedectomies in patients with otosclerosis, as the latter may introduce mechanical trauma to the inner ear and cause post-operative dizziness.^1^^,^^8^ Our study does not demonstrate a statistically significant difference in the rate of dizziness, with both MP and RL patients having similarly low rates of post-operative dizziness requiring medication. However, given the variability in operative experience and surgical approach, additional research is needed to isolate more accurately the contribution of the surgical approach versus the prosthesis type. Importantly, no patients in our cohort required long-term management of dizziness concerns, highlighting the overall safety of both approaches.

Despite this, if we use the immediate post-operative medication regimen as a proxy for dizziness severity (per surgeon regimen, diazepam was prescribed to patients with more severe dizziness and meclizine for those with mild dizziness), the increased prescription of diazepam for patients receiving stapedectomies suggests that severity of post-operative dizziness for RL stapedectomies may be greater compared MP stapedotomies. With this said, the relatively small number of dizzy patients makes it difficult to discern whether this statistical significance would translate to clinical significance. Additional work is needed to verify this finding.

Benzodiazepines have been proven beneficial in short-term use of less than one month and are therefore an acceptable treatment for post-operative dizziness following stapes surgery which was shown in our study to typically improves after one week. However, these medications can be highly addictive, can worsen pre-existing conditions such as anxiety or insomnia and can increase the likelihood of accidental injuries, emergency department visits and hospital admissions and, therefore, should be used sparingly.^19^^,^^20^ This information suggests that pre-operative consideration for possible post-operative benzodiazepine use should be properly considered and discussed with patients. While the overall rate of benzodiazepine use was low, it was mildly higher in the stapedectomy group.

### Limitations

Our study has several limitations. First, as a retrospective analysis, we could not control for possible confounding variables such as patient underlying vestibular weakness or predisposition for dizziness. Due to the retrospective nature of the study the presence of dizziness was determined by either admission or the administration of diazepam or Meclizine, which may have excluded patients who experienced milder dizziness and did not request medication or refused treatment. Also, dizziness severity was estimated by rates of diazepam prescription as opposed to meclizine, which may be confounded by details such as patient-specific vertigo tolerance or surgeon-specific diazepam prescription rates. We understand this is an imperfect measurement of dizziness, an outcome that is typically hard to systematically define retrospectively. Future work in this field would benefit from prospective trials of the two surgical methods.

Additional multi-institutional prospective studies are needed that could address these limitations, particularly the limitation of surgeon preference. It is important to note that, in stapes surgery, surgeons usually have a strong preference for technique and prosthesis; therefore, conducting a randomized controlled study, controlling for prosthesis type would be very difficult. Instituting a multi-institutional study would address the limitation of sample size and allow for more surgeons with varying experience utilizing a specific prosthesis to be evaluated which will allow controlling for confounding variables which can affect hearing outcomes and complication rate. Future studies can also seek to assess prosthesis and hearing outcomes improvements over time.

- Condition and treatments: Otosclerosis causes abnormal bone growth at the stapes footplate, impairing sound transmission. Standard surgeries are stapedotomy and stapedectomy. Post-operative dizziness is common; severe cases may be treated with benzodiazepines.
- Prior evidence: Many studies compare hearing outcomes—typically air–bone gap (ABG) closure—between procedures. Most report larger improvements with stapedotomy, though results are mixed and debated.
- Cohort: Retrospective institutional series of 276 ears (144 stapedotomies, 132 stapedectomies).
- Hearing outcomes: No significant difference in ABG closure between procedures; stapedectomy showed a larger improvement in speech recognition threshold (SRT). Overall, both procedures produced clinically meaningful hearing gains.
- Vestibular/medication outcomes: Post-operative dizziness rates did not differ by procedure, but benzodiazepines were prescribed more often after stapedectomy—a point to consider in pre-operative counseling and expectations.

## Conclusion

Our study contributes to the existing literature on hearing outcomes and post-operative complications associated with different stapes prosthesis types, using a large surgical cohort. Our findings suggest that, while audiologic outcomes are similar after both stapedotomy and stapedectomy, hearing outcomes were, if anything, slightly better in the stapedectomy group. Additionally, while stapedectomy may be associated with increased benzodiazepine use for managing immediate post-operative dizziness (with no long-term dizziness observed for either technique), it does not appear to be an inferior technique. These findings highlight the importance of understanding the benefits and potential drawbacks of each surgical approach and stapes prosthesis option, which can better inform patient counselling and decision-making for stapes surgery.
